# Protective Role of Rosmarinic Acid in Experimental Urolithiasis: Understanding Its Impact on Renal Parameters

**DOI:** 10.3390/ph17060702

**Published:** 2024-05-29

**Authors:** Anelise Felício Macarini, Luísa Nathalia Bolda Mariano, Mariana Zanovello, Rita de Cássia Vilhena da Silva, Rogério Corrêa, Priscila de Souza

**Affiliations:** Programa de Pós-Graduação em Ciências Farmacêuticas, Universidade do Vale do Itajaí, Rua Uruguai 458, Itajaí 88302-901, Brazil

**Keywords:** ammonium chloride, calcium oxalate, crystalluria, ethylene glycol, phenolic compound

## Abstract

This study aimed to assess the ability of rosmarinic acid (RA) to prevent kidney stone formation in an ethylene glycol and ammonium chloride (EG/AC) model. There was an increase in diuresis in the normotensive (NTRs) and hypertensive rats (SHRs) treated with hydrochlorothiazide (HCTZ) and exposed to EG/AC, while RA restored urine volume in NTRs. The EG/AC groups exhibited lower urine pH and electrolyte imbalance; these parameters were not affected by any of the treatments. Both HCTZ+EG/AC and RA+EG/AC reduced calcium oxalate crystal formation in NTR and SHR urine. Kidney tissue analysis revealed alterations in oxidative stress and inflammation parameters in all EG/AC-receiving groups, with RA enhancing antioxidant defenses in SHRs. Additionally, crystals were found in the kidney histology of all EG/AC-exposed groups, with reduced Bowman’s capsule areas in NTRs and SHRs. The NTR VEH+EG/AC group showed intense renal damage, while the others maintained their structures, where treatments with HCTZ and RA were fundamental for kidney protection in the NTRs. Docking analysis showed that RA exhibited good binding affinity with matrix metalloproteinase-9, phosphoethanolamine cytidylyltransferase, and human glycolate oxidase enzymes. The data disclosed herein underscore the importance of further research to understand the underlying mechanisms better and validate the potential of RA for clinical use.

## 1. Introduction

Nephrolithiasis is the process of forming kidney stones, also called renal calculi. It is a painful and often recurrent condition that can affect all genders, races, and ages, despite being more common in white males [1,2]. Renal stones can be either infectious or non-infectious, caused by genetic defects or even an adverse effect of drugs [3]. The non-infectious stones can be composed of calcium oxalate, calcium phosphate, uric acid, and ammonium urate. Identifying the composition of the stones is important for diagnosis and further management or treatment [3]. This study primarily focuses on two clinically significant calculi including calcium oxalate monohydrate (whewellite, CaC_2_O_4_·H_2_O) and calcium oxalate dihydrate (weddellite, CaC_2_O_4_·2H_2_O) [3,4]. In addition to the considerable burden on patients, nephrolithiasis also poses a substantial financial strain on the healthcare system [5].

Associations have been found linking renal stones and weight gain (35-pound gain since early adulthood), body mass index (BMI, higher than baseline), and diabetes mellitus (independent of diet and body size) [1]. There are also non-dietary risk factors such as family history, history of gout, living or working in a hot environment, and some systemic diseases such as primary hyperparathyroidism, renal tubular acidosis, hypertension, cardiovascular, and Crohn’s disease [1,6].

As previously mentioned, nephrolithiasis is a multifactorial disease that requires an accurate diagnosis to initiate appropriate treatment promptly. A diagnosis can be made through images (X-ray, ultrasound, or computed tomography), biochemical urine and blood tests, and analyzing the mineral composition of a passed stone [3]. Treatment should be individualized for each patient, considering the size, number, location, constitution, morphology, hardness, and mobility of the stone [3]. When present, renal colic is treated with non-steroidal anti-inflammatory drugs (NSAIDs), opioids, or anesthetics (e.g., ketamine). If spontaneous stone passage is not expected to occur, ureteroscopy, shock wave lithotripsy, or percutaneous nephrolithotomy should be considered [3].

Considering the frequent recurrence of stones, the primary emphasis on preventing their reappearance involves adjusting lifestyle, dietary habits, and fluid intake. In some instances, pharmacological treatment might be contemplated [3]. Specifically for calcium oxalate stones, key pharmacological options include citrates, allopurinol, calcium, magnesium, sodium bicarbonate, pyridoxine, and hydrochlorothiazide [3]. Furthermore, within cultural practices, a diverse array of plant-based therapy is employed to prevent lithogenesis, by enhancing diuresis, alleviating pain, or facilitating natural elimination by the body. This utilization can be attributed to the presence of numerous bioactive compounds in these plants, including polyphenols, flavonoids, phytosterols, saponins, furanochromones, alkaloids, and terpenoids [7]. 

Lamiaceae herbs, such as *Orthosiphon stamineus* (Java Tea) [8,9] and *Glechomae herba* [10], are utilized in different regions of the world for kidney and bladder stones, antiurolithic activity, cleansing, and other purposes [8,9,10]. Rosmarinic acid is a phenolic acid highly found in those plants [8,9,10,11], but its isolated form has received limited research attention in relation to renal stones. In addition to its antioxidant, antibacterial, anti-inflammatory, and other activities, rosmarinic acid has also been discovered to reduce tubular dilatation, Bowman’s capsule enlargement, degradation of the tubular epithelium, and localized glomerular necrosis in a kidney ischemia model [7,11]. Based on these considerations, we propose a kidney stone model treated with rosmarinic acid to elucidate its impact on normotensive (NTRs) and spontaneously hypertensive rats (SHRs).

## 2. Results

### 2.1. Weight Loss in the NTR and SHR Animals in the Period of the Experiment

The animals were monitored throughout the experiment and variations in body weight were detected, especially in the groups that received ethylene glycol and ammonium chloride (EG/AC). At the end of the experiment (within 10 days), the weight loss of the untreated vehicle group was approximately 7% in NTRs, while no loss was observed in the respective SHR group. Although treatments with hydrochlorothiazide (HCTZ) or rosmarinic acid (RA) were promising in some parameters, which will be discussed below, they were not able to attenuate this weight loss.

### 2.2. Six-Hour Diuresis

**Figure 1:**
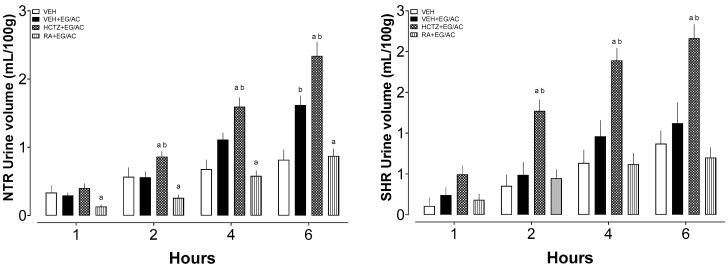
Urine volume of the NTR and SHR groups. a: *p* < 0.05 compared with the VEH+EG/AC group; b: *p* < 0.05 compared with the VEH group.

Immediately after the last day of treatment (10 days), the diuresis experiment was conducted (Figure 1). In NTRs, it can be observed that HCTZ+EG/AC group presented increased diuresis from the second hour onward when compared with both the VEH and VEH+EG/AC groups. The RA+EG/AC group remained as the VEH group, showing that it restored the initial conditions of the animals, possibly preventing the damage caused by EG/AC ingestion. In SHRs, only the HCTZ+EG/AC group showed an increase in diuresis from the second hour onward when compared with both the VEH and VEH+EG/AC groups.

**Figure 2:**
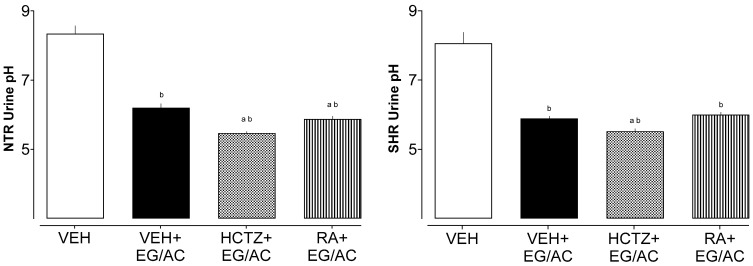
Urine pH of the NTR and SHR animals. a: *p* < 0.05 compared with the VEH+EG/AC group; b: *p* < 0.05 compared with the VEH group.

As expected, the urine pH of the groups receiving EG/AC was lower than the VEH (receiving only regular water) because of the chemical aspects of these compounds (Figure 2). The treatments with HCTZ or RA did not modify this parameter. 

### 2.3. Crystalluria in Urine

**Figure 3:**
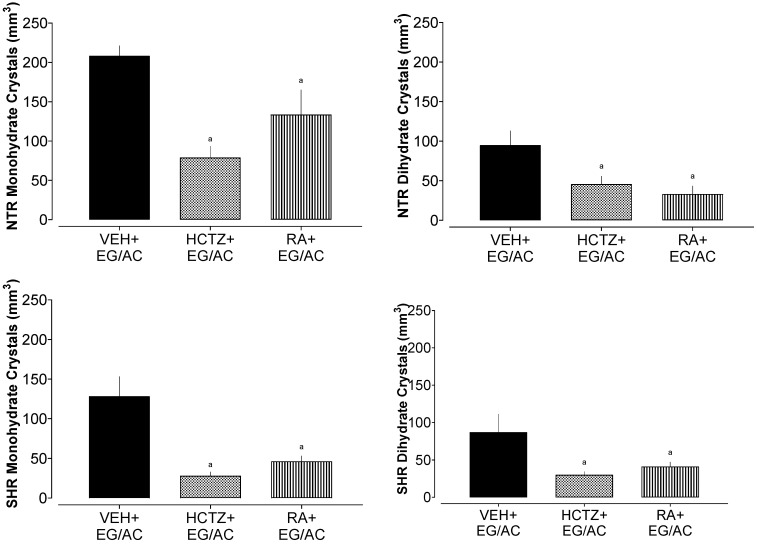
Crystals in the urine of the NTR and SHR groups treated with HCTZ or RA. a: *p* < 0.05 compared with the VEH+EG/AC group.

**Figure 4:**
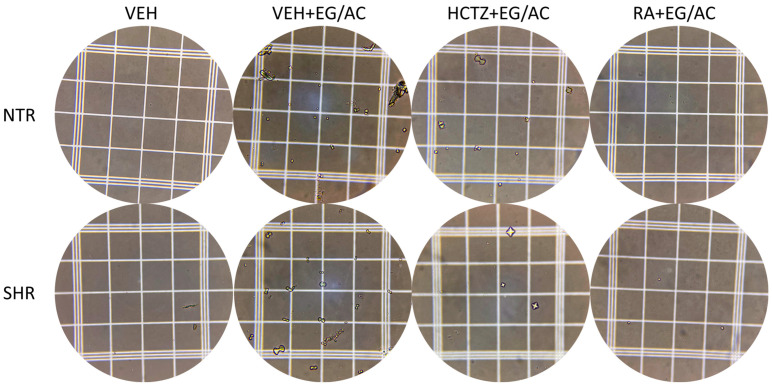
Representative images of the urine from the NTR and SHR groups.

An aliquot of the urine collected from the diuresis experiment was utilized to observe the formation, identification, and counting of crystals under the microscope, as depicted in Figure 3 and Figure 4. An intensive formation of calcium oxalate crystals was observed in the untreated NTR and SHR groups (VEH+EG/AC), as anticipated from the utilized model. Both the HCTZ+EG/AC- and RA+EG/AR-treated groups were effective in diminishing the formation of crystals, of both formats, when compared with the VEH+EG/AC group, suggesting the ability of these treatments to prevent urolithiasis. 

### 2.4. Electrolytes and Biochemical Parameters in Urine and Serum

Regarding the ion’s excretion in the urine shown in Table 1, we first detected a very significant difference between the VEH (untreated) and VEH+EG/AC groups, especially with reductions in the excretion of Na^+^ and K^+^. The group treated with HCTZ showed increased electrolyte values when compared with VEH+EG/AC, confirming its ability to stimulate urine volume and saluresis. The groups treated with RA did not show significant changes when compared with VEH+EG/AC, corroborating what had already been identified in the analysis of urine volume. These data suggest that although RA treatment was effective in reducing crystal formation, it probably does not share diuretic and/or saluretic action for this purpose.

In addition to ion excretion, variations were noted in urea and creatinine elimination, with the NTR VEH+EG/AC group displaying reduced urea excretion and the SHR VEH+EG/AC group demonstrating diminished creatinine excretion. The glucose content detected in the urine was low in all groups, with changes only in the groups treated with HCTZ+EG/AC.

Regarding the serum analysis shown in Table 2, although not all the results are statistically significant, when analyzing the averages obtained between VEH and VEH+EG/AC, an increase in electrolytes (especially Na^+^ and K^+^) in plasma is noted, which could explain the reduction in these ions in urine described in the previous table. The groups that received RA showed discrete and non-significant changes in most of the markers analyzed. The group that received HCTZ showed changes in several markers, highlighting the significant reduction in glucose and cholinesterase in the NTR animals.

### 2.5. Oxidative Stress and Inflammation Parameters

The renal and livers of the animals were used to assess oxidative stress and inflammation parameters. In the kidney tissue (Table 3), both the NTR and SHR groups exposed to EG/AC showed changes in the analyzed parameters; however, the group with the greatest changes was the NTR group that received treatment with HCTZ, showing increased values of reduced glutathione (GSH) and decreased activities of inflammatory markers myeloperoxidase (MPO) and N-acetyl-beta-D-glucosaminidase (NAG). Furthermore, the SHR group exposed to EG/AC, and which received treatment with RA, showed increased values in the activity of the enzymes glutathione-S-transferase (GST), superoxide dismutase (SOD), and catalase (CAT), indicating a possible reinforcement in endogenous antioxidant defenses.

The changes in liver tissue (Table 4) were more evident when compared with those obtained with the kidney samples. Interestingly, both treatments were able to restore depleted values of oxidative markers seen in samples from the EG/AC group that received only VEH. Furthermore, we observed that the NTR groups that received EG/AC showed a reduction in the enzymatic activity of inflammatory markers, while in the SHR groups, this change was not significant.

### 2.6. Histological Analysis

**Figure 5:**
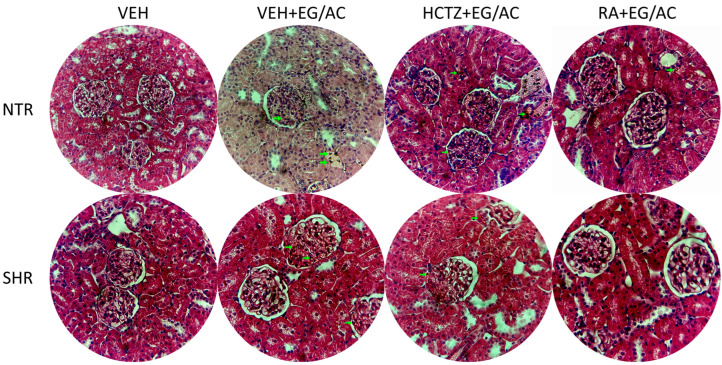
Histological images of kidney tissue at 40× augmentation.

**Figure 6:**
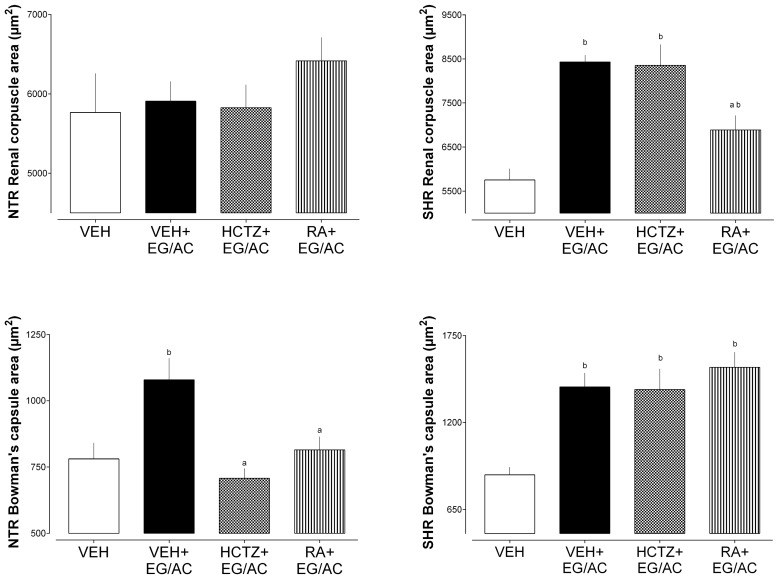
Renal corpuscle areas and Bowman’s capsule areas measured at 40× augmentation in both animal groups. a: *p* < 0.05 compared with the VEH+EG/AC group; b: *p* < 0.05 compared with the VEH group.

Crystals were found in the kidney sections of all groups exposed to VEH+EG/AC in both the NTR and SHR models. Qualitatively, it is noted that the NTR VEH+EG/AC group showed intense renal damage, while the others maintained their structures, where treatments with HCTZ and RA were fundamental for the NTR group. In NTRs, the HCTZ+EG/AC and RA+EG/AC groups displayed significantly smaller Bowman’s capsule areas compared with the VEH+EG/AC group. Similarly, in the SHR animals, the RA+EG/AC group exhibited reduced Bowman’s capsule areas relative to the VEH+EG/AC group. Moreover, regarding renal corpuscle areas, SHR tissues presented augmented renal corpuscle compared with the VEH group. Only the group treated with RA presented reduced area when compared to the VEH+EG/AC group. All the groups receiving EG/AC exhibited high values of Bowman’s capsule areas (Figure 5 and Figure 6). 

### 2.7. Molecular Docking

**Figure 7:**
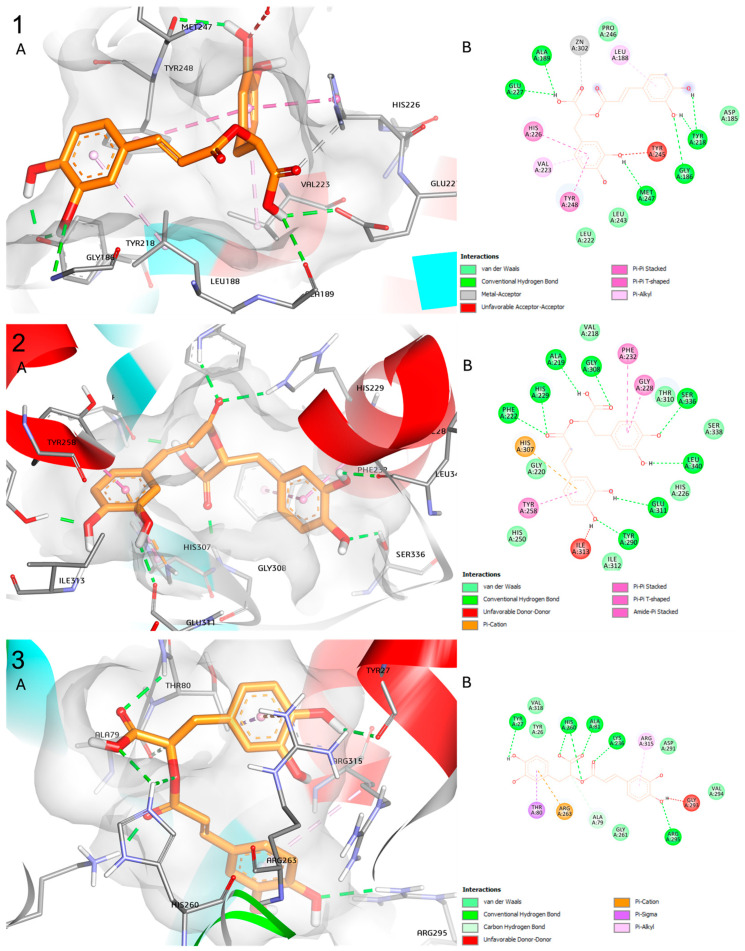
Docking images of the predicted binding interaction between RA and MMP-9 (**1**), PC (**2**). and GO (**3**). The 3D binding interactions (**A**) and 2D binding interactions (**B**) are represented below.

In the docking analysis, we observed that RA presented a good binding affinity (lower than −7.0 kcal/mol) in three of the four enzymes analyzed. The best affinity, as shown in Table 5, is with matrix metalloproteinase (MMP)-9, followed by phosphoethanolamine cytidylyltransferase (PC), and human glycolate oxidase (GO). Docking images of the predicted binding interaction between RA and the enzymes are depicted in Figure 7. 

## 3. Discussion

The model chosen to induce crystalluria in the rats was ethylene glycol (EG) and ammonium chloride (AC) 1%, which was continuously administered in the drinking water. Khan 1997 [12] stated that after the chronic administration of EG 0.75% to male Sprague-Dawley rats, the urinary excretion of oxalate increased rapidly, concomitant with a decreased excretion of calcium, magnesium, and citrate. The combined treatment with EG+AC resulted in crystalluria, present in all rats by the third day, followed by nephrolithiasis in the seventh day. It is important to highlight that in female rats, the EG treatment in association with urine acidification produced urine crystalluria, but crystal deposits in the kidney were only observed in male rats [12]. The proposed mechanism of action for EG-induced renal toxicity in animals involves its metabolism into oxalic acid. This leads to the precipitation and growth of insoluble calcium oxalate crystals within the renal tubule epithelium, which becomes concentrated in the kidneys before being cleared in urine [13]. Prolonged, high-level exposure to EG can disrupt the equilibrium between the formation and clearance rates of oxalic acid, potentially causing an accumulation of calcium oxalate crystals in renal tubule epithelial cells. This results in the degeneration of renal tubules, likely attributable to physical injury or localized oxidative stress, predominantly affecting the proximal tubule but potentially extending into the renal pelvis [13]. 

In humans the formation of renal stones results from an increase in urinary supersaturation, leading to the creation of crystalline particles. While most of these particles are excreted freely and do not signify symptomatic stone disease, the retention of solid particles within the kidney can facilitate their growth into full-size stones [14]. Crystals can be retained at various kidney sites and undergo growth and aggregation. Stone formation requires not only crystal retention but also stone positioning in areas where they can cause papillary surface ulceration and create a stone nidus, with renal tubular injury playing a crucial role in this process [14]. Additionally, renal tubular injury promotes crystal nucleation at low supersaturation and enhances crystal–cell interactions. Abnormal particle retention is essential for kidney stone formation, as the internalized crystals in the interstitium grow and aggregate, ultimately developing into renal stones [14].

In our experiments, we observed mortality in the VEH+EG/AC group, especially in the NTR group. This lethality was also observed by Bervinova et al., 2022 [15], where 13 out of 30 Sprague-Dawley rats died while receiving EG 1%. Although the EG-induced kidney stone model simplifies the complex factors that contribute to kidney stone formation in humans, such as genetics, dietary habits, and various metabolic conditions, it can provide valuable insights into some aspects of kidney stone formation and pathogenesis, making it a useful tool for research.

The presence of nephrolithiasis has been associated with hypertension in clinical studies, suggesting that there may be a connection between these two conditions. One potential link is the increased urinary calcium excretion often observed in individuals with hypertension. This heightened calcium excretion could serve as a pathogenic factor contributing to the development of kidney stones [16]. Cappuccio et al., 1999 [17], stated that hypertension in middle-aged men can be considered a predictor of kidney stone disease rather than being a consequence of renal damage caused by pre-existing kidney stones. Therefore, employing both NTR and SHR animals in the EG+AC kidney stone model tested is of clinical relevance. This relevance is further supported by the observation that SHRs of the Okamoto–Aoki strain [18] that received a renal transplant from compatible normotensive rats normalized the arterial pressure [19], indicating that the primary factors contributing to hypertension in SHR are associated with their kidneys or that the kidneys received can compensate for other factors that contribute to increased blood pressure in SHRs.

Diuretics, such as hydrochlorothiazide, are used in the management of hypertension to reduce blood volume, thereby lowering peripheral vascular resistance. Hydrochlorothiazide is also clinically employed in the prevention of kidney stone recurrence. However, a recent double-blind, randomized clinical trial found that the incidence of kidney stone recurrence in patients receiving daily doses of 12.5 mg, 25 mg, or 50 mg of hydrochlorothiazide did not differ significantly from those receiving a placebo [20]. Nevertheless, diuretics still play a vital role in increasing urine volume and reducing urine supersaturation, facilitating the clearance of crystals.

To our knowledge, this is the first in vivo study to evaluate the anti-urolithic effect of isolated rosmarinic acid (RA). The dose of 3 mg/kg used in the present study was selected based on previously published data obtained from an acute diuretic activity experiment.

In the aforementioned study, RA was administered to rats at doses of 0.3, 1, and 3 mg/kg. Among these doses, the 3 mg/kg dose of RA demonstrated significant effects on urine volume and urinary parameters compared with the vehicle-treated group, indicating its effectiveness as a diuretic agent [21]. In the results described herein, although RA did not show increased diuresis, it prevented the formation of mono and dihydrate calcium oxalate crystals in the urine of the NTR and SHR animals. In an in vitro study, RA presented the potential to reduce monohydrate CaOx crystals in synthetic urine, but not dehydrate crystals [21]. Another in vitro study evaluated *Orthosiphon stamineus* standardized water extract, where the main constituent is RA, and its capability as a chemolytic agent. They collected CaOx stones and accessed the dissolution power of the extract at pH 5 for 8 weeks. They observed a reduction of approximately 69% in CaOx stone size, where potassium citrate (positive control) presented a 40% reduction in stone size, along with a morphology change. Microscopic pictures of CaOx stone taken pre-experiment showed hard stones with irregular surfaces, and in the post-experiment, the stones presented more regular surfaces and exposed the interior layer in a well-arranged pattern [22]. In an in vivo study, a phenolic-rich extract containing RA diminished CaOx crystals, induced by EG/AC in both quantity and size [23].

Our results showed no difference in the diuresis of the animals in the group that received RA treatment. The data suggest that the beneficial effect of RA in the urolithiasis model studied herein is independent of the increase in urinary volume, although, without the induction of the disease, this bioactive behaves differently. Moser et al., 2020 [21], reported that after the administration of rosmarinic acid to male Wistar rats, it presented a diuretic and natriuretic effect at a 3 mg/kg dose, besides providing a K^+^ and Ca^2+^ sparing effect.

Regarding serum electrolytes, minor differences were found when comparing the VEH and VEH+EG/AC groups, with only an increase in Na^+^ in the serum of the NTR VEH+EG/AC group. Green et al., 2005 [24], also found no difference in the Na^+^, K^+^, and Cl^−^ content in the serum of male Sprague-Dawley rats that received treatment with 0.75% of EG for 4 weeks when compared with a control group that did not ingest EG, nor did it affect the acid–base chemistry of rats with normal renal function. However, in another study, also using EG/AC-induced urolithiasis, an increase in serum Na^+^, K^+^, Ca^2+^, and Cl^−^ was found, along with urea, creatinine, uric acid, and phosphorus in the lithiac group control (receiving only EG/AC with no other treatment) [23]. This difference regarding our finding can be due to the animals used as well as the male/female difference in response to the EG/AC treatment.

The histological analysis of the kidneys presented some findings. Renal corpuscle degeneration, and sometimes the absence and presence of inflammatory cells and blood infiltration, was already observed with exposure to EG [25,26]. In addition, disrupted renal parenchyma can also be found, with loss of structural arrangement of the renal tubules and focal calcification of glomeruli-tubular structures [26], along with tubular dilatation/degeneration and intratubular crystals [25].

These histological findings indicate that RA protects the kidney against damage caused by the metabolites of the EG treatment. Renal lithiasis is known to augment the intensity of oxidative stress in patients with this condition when compared with healthy individuals, presenting an accumulation of malonic aldehyde—a product of lipid peroxidation, a lowering of protein–SH groups, and, in compensation, an augment of non-enzymatic antioxidant level [27]. In the renal tissue of NTR animals, the most significant change observed was diminished catalase (CAT) activity in the EG/AC-treated group. In chronic inflammation and oxidative stress overload, tissue pH can be lowered, compromising CAT activity [28]. Upon administration of 1.25–2.5% ethylene glycol (EG) to albino Sprague-Dawley rats, a decrease in catalase (CAT) activity was observed in both the kidney and liver of the animals, which can be explained by the gradual exhaustion of this enzyme [29], although other studies show an increase in CAT activity in the kidney of hyperoxaluric rats [30]. GST and GSH were unaffected by EG exposure, while SOD activity was diminished in the liver and augmented in the kidney [29]. These results are partly in accordance with our findings, although the specimen and period were different.

As aforementioned, EG is metabolized in the liver. In the process, one step of the reaction that transforms ethylene glycol into oxalic acid is the oxidation of glycolic acid to glyoxylic acid [31] by a peroxisomal enzyme called glycolate oxidase (GO) [32,33]. Previous docking studies analyzed some flavonoids, with in vitro antilithic activity, and verified they presented a good affinity to GO [33,34] in silico. Other key players in lithogenesis are the matrix metalloproteinases (MMP)-2 and MMP-9. These endopeptidases are involved in the growth of renal stones by breaking down collagen and fibronectin, violating urinary tract barriers. In urolithiasis, ROS content generated from calcium oxalate stone injures and activates NF-κB, leading to MMP-9 transcription. This MMP-9 upregulation deteriorates the urothelium, promoting stone growth. Additionally, MMP-9 and MMP-2 are implicated in renal fibrogenesis, worsening kidney function in stone formers [34]. In accordance with this study, we also observed more affinity to MMP-9 than MMP-2.

Aggarwal et al. [35] isolated five proteins from the matrix of human CaOx kidney stones. Two of them are promoters, two inhibitors, and one with dual activity. One of the promoters is phosphoethanolamine cytidylyltransferase, likely playing a crucial role in promoting kidney stone formation by contributing to the synthesis of phosphatidylethanolamine (PtdEtn), which is found in the lipid content of kidney stones. The synthesis of PtdEtn from ethanolamine generates glyoxal, which is involved in the synthesis of oxalic acid, suggesting a potential link between this enzyme and stone formation [32,35].

The gastric passage of RA can lead to a significant reduction in its concentration because of the acidic gastric environment. The form of intake can affect RA’s passage through the gastrointestinal tract. An in vitro study suggested that hard gelatine capsules can protect RA from the process of gastrointestinal digestion [36]. In the body, the absorption of RA occurs via enterocytes by paracellular transport in tight junctions, but it is suggested, in both in vitro and in vivo investigations, that its intestinal permeability is approximately 1% of the applied dose. Also, it is mostly insoluble in water, so it is distributed throughout the circulatory system via protein carriers [36]. After peritoneal administration, RA was predominantly found in the kidneys, suggesting that they are responsible for its metabolism, followed by the lungs, spleen, and liver. The levels of RA in the brain remained low, indicating that it does not permeate the blood–brain barrier [37].

Rosmarinic acid possesses many pharmacological activities already unraveled, and we can add yet another one to this list, even though more studies must be performed to enhance our understanding of this promising compound. In addition, rosmarinic acid exhibits a safety profile across various in vitro toxicity studies [38,39,40,41]. In two clinical studies, no adverse self-reported events were noted, and routine blood tests revealed no significant abnormalities [42,43]. However, continued research and monitoring are essential to ensure its safety, particularly when considering its use in long-term treatments or higher dosages.

## 4. Methods and Materials

### 4.1. Products

Rosmarinic acid, ethylene glycol, ammonium chloride, and hydrochlorothiazide were obtained from Sigma-Aldrich Chemical Co. (St. Louis, MO, USA).

### 4.2. Animals

Female normotensive (NTRs) and spontaneously hypertensive rats (SHRs), aged 12 to 14 weeks, were sourced from the University of Vale do Itajaí bioterium following the approval of the institutional ethics committee (authorization nº 013/21). The animals were housed in a controlled environment with a 12 h light/dark cycle, maintained at a temperature of 22 ± 2 °C, and provided with ad libitum access to food and water.

### 4.3. Animal CaOx Crystallization Model

The experiment involved administering water ad libitum containing ethylene glycol (EG) and ammonium chloride (AC) at a concentration of 1% each to induce the formation of calcium oxalate (CaOx) crystals. In a male rat model, the administration of 0.75% EG aqueous solution increased oxalate and CaOx supersaturation in urine while decreasing the excretion of calcium, magnesium, and citrate within 10 days [12]. At this concentration, EG produced kidney stones in males, but only CaOx crystallization in females, where the formation of kidney stones required solutions of 1% or higher [12].

The NTR and SHR animals were divided into four groups each. One group had regular drinking water access (vehicle; VEH). The three other groups received EG+AC 1% in the drinking water plus the treatments (once a day; for 10 days) with hydrochlorothiazide at 5 mg/kg (HCTZ+EG/AC), rosmarinic acid at 3 mg/kg RA+EG/AC, or just vehicle (VEH+EG/AC).

### 4.4. Diuresis

For the diuresis assay, the animals were individually placed in individual metabolic cages, and the urine volume was collected over a 6-h period. The volume was calculated in relation to body weight and expressed as mL/100 g [21,44,45,46]. Electrolyte excretion (Na^+^, K^+^, Ca^2+^, and Cl^−^), pH, conductivity, creatinine, urea, and glucose were measured in each urine sample at the end of the experiment. Also, crystals (mono and dihydrate) were counted using a Neubauer chamber.

#### 4.4.1. pH and Conductivity Assessment in Urine

A digital conductivity and pH meter (model 86505, AZ Instrument Corp., Taichung City 427, Taiwan) was used to measure conductivity and pH levels.

#### 4.4.2. Electrolytes and Biochemical Concentration in Urine

The levels of Na^+^ and K^+^ in the urine were assessed using a flame photometer (BFC-300 by Benfer, São Paulo, Brazil). In brief, the equipment underwent calibration with a standard solution in accordance with the manufacturer’s instructions. Between each measurement, deionized water was added until the reading returned to the baseline zero value.

Colorimetric tests were employed to assess the urinary levels of Cl^−^, Ca^2+^, urea, creatinine, and glucose (Bioclin, Belo Horizonte, MG, Brazil) in accordance with the manufacturer’s guidelines.

### 4.5. Biochemical, Oxidative Stress, and Inflammation Parameters

Immediately after the 6-h diuresis experiment, the rats were anesthetized (xylazine 10 mg and ketamine 80 mg/kg) and their blood, kidneys, and liver were collected.

Blood was centrifuged to separate the serum and electrolyte content (Na^+^, K^+^, Ca^2+^ and Cl^−^). Creatinine, urea, glucose (measured as stated in Section 4.4.2), oxaloacetic transaminase (TGO), pyruvic transaminase (TGP), and cholinesterase were measured using colorimetric tests (Bioclin, Belo Horizonte, MG, Brazil) in accordance with the manufacturer’s guidelines.

The organs were put in phosphate buffer and homogenized in potassium phosphate buffer (200 mM with pH 6.5; 1:3 weight/volume) by maceration. With this homogenate, reduced glutathione (GSH) and lipid hydroperoxide (LPO) content were measured. After that, the homogenate was centrifuged, and superoxide dismutase (SOD), glutathione-S-transferase (GST), and catalase (CAT) activities were measured with the supernatant. The precipitate was resuspended with buffer (HTAB 0.5%), and myeloperoxidase (MPO) and N-acetylglucosamine (NAG) activity were evaluated. The methodology was performed following a previously mentioned method [47].

### 4.6. Histological Analysis

The histological sections of the kidney were stained with hematoxylin–eosin. Histological analyses were conducted on the left kidney, and images were captured to measure the Bowman’s capsule area. Utilizing ImageJ 1.38e [48] software, the areas of the renal corpuscle were measured (external capsule and glomerulus area). The final measurement of the Bowman’s capsule was obtained by subtracting the external capsule area from the glomerulus area. Random renal corpuscles were measured with approximately 10–15 measures per group.

### 4.7. Statistical Analysis

The data were presented as mean ± S.E.M, and statistical analysis was performed using one or two-way ANOVA followed by Dunnett’s multiple comparisons test using GraphPad Prism version 8.0.1 for Windows (GraphPad Software, Boston, MA, USA). Statistical significance was determined at a *p*-value of less than 0.05.

### 4.8. Molecular Docking

Crystal structures of human glycolate oxidase (2RDT), the MMP-2 catalytic domain (1HOV), the MMP-9 catalytic domain (4XCT) [34], and phosphoethanolamine cytidylyltransferase (3ELB) [32] were retrieved from Protein Data Bank (PDB) [49].

The proteins were prepared using the AutoDockTools (ADT 1.5.7) [50]: co-crystals ligands, water, and ions were removed, and hydrogens and Kollman charges were added. The 3D structure of the ligand (rosmarinic acid) was retrieved from PubChem [51] in SDF format, converted to MOL2 format using OpenBabel GUI [52], and the structure was minimized with UCSF Chimera 1.17.3 [53]. Molecular docking was performed using AutoDock Vina [54], with the exhaustiveness of the global search set to 30. All 3D and 2D images were generated with the BIOVIA Discovery Studio program.

## 5. Conclusions

In conclusion, this study demonstrates the potential of RA in preventing kidney stone formation in a model induced by ethylene glycol and ammonium chloride. RA effectively restored urine volume in NTRs and reduced calcium oxalate crystal formation in both NTRs and SHRs. RA treatment enhanced antioxidant defenses and mitigated oxidative stress and inflammation in SHRs. Histological analysis revealed that RA treatment preserved kidney structure and prevented the severe renal damage seen in the NTR disease model. Additionally, RA exhibited strong binding affinities with key enzymes involved in kidney stone formation, suggesting its therapeutic potential. These findings underscore the importance of further research to elucidate the mechanisms of RA and validate its clinical application for kidney stone prevention.

## Figures and Tables

**Table 1:** Electrolytes and biochemical parameters in the urine of the NTR and SHR groups.

	Group	Na^+^ (mmol/L)	K^+^ (mmol/L)	Cl^−^ (mmol/L)	Ca^2+^ (mg/dL)	Urea (mg/dL)	Creatinine (mg/dL)	Glucose (mg/dL)
**NTRs**	VEH	94.00 ± 16.73	57.12 ± 19.76	85.14 ± 3.51	11.91 ± 0.48	7.95 ± 1.84	19.26 ± 10.75	19.50 ± 4.60
	VEH+EG/AC	32.78 ± 11.49 ^b^	24.95 ± 17.46 ^b^	89.75 ± 9.11	6.28 ± 3.28 ^b^	3.68 ± 0.78 ^b^	13.23 ± 3.82	18.93 ± 5.01
	HCTZ+EG/AC	75.00 ± 27.37 ^a^	54.00 ± 11.86 ^a^	121.45 ± 3.35 ^a,b^	11.32 ± 0.89 ^a^	6.63 ± 3.4 ^a^	7.27 ± 3.08 ^b^	13.92 ± 0.85 ^a,b^
	RA+EG/AC	31.67 ± 19.58 ^b^	18.40 ± 16.63 ^b^	83.98 ± 22.86	6.26 ± 3.36 ^b^	5.84 ± 1.67	22.30 ± 7.34 ^a^	22.54 ± 4.33
**SHRs**	VEH	120.00 ± 36.51	67.04 ± 12.11	81.68 ± 11.37	12.19 ± 0.15	5.07 ± 1.76	27.25 ± 5.52	23.52 ± 2.40
	VEH+EG/AC	53.33 ± 30.80 ^b^	45.93 ± 15.81 ^b^	119.07 ± 8.75 ^b^	12.46 ± 0.40	5.94 ± 2.06	17.12 ± 4.99 ^b^	23.27 ± 4.27
	HCTZ+EG/AC	124.00 ± 31.62 ^a^	39.60 ± 10.66 ^b^	120.69 ± 7.95 ^b^	11.81 ± 1.60	7.12 ± 1.80	8.20 ± 2.47 ^a,b^	18.49 ± 1.88 ^a,b^
	RA+EG/AC	40.50 ± 14.09 ^b^	35.90 ± 13.19 ^b^	88.82 ± 10.00 ^a^	7.82 ± 1.78 ^a,b^	3.07 ± 0.59 ^a^	20.48 ± 6.27 ^b^	22.65 ± 1.48

Values expressed as mean ± standard deviation; ^a^: *p* < 0.05 compared with the VEH+EG/AC group; ^b^: *p* < 0.05 compared with the VEH group.

**Table 2:** Electrolytes and biochemical parameters in the serum of the NTR and SHR groups.

	Group	Na^+^ (mmol/L)	K^+^ (mmol/L)	Cl^−^ (mmol/L)	Ca^2+^ (mg/dL)	Urea (mg/dL)	Creatinine (mg/dL)	Glucose (mg/dL)	TGO (U/L)	TGP (U/L)	Colinesterase (U/L)
**NTRs**	VEH	174.00 ± 27.02	4.20 ± 0.84	68.69 ± 5.75	9.90 ± 0.86	25.17 ± 9.60	2.71 ± 0.22	171.15 ± 5.41	11.70 ± 0.58	2.36 ± 0.25	9585 ± 5179
	VEH+EG/AC	230.00 ± 29.66 ^b^	6.22 ± 0.91	72.51 ± 4.09	9.57 ± 0.67	17.99 ± 6.46	2.67 ± 0.60	139.29 ± 22.30 ^b^	6.70 ± 3.06 ^b^	2.43 ± 0.38	16,514 ± 5772
	HCTZ+EG/AC	202.50 ± 26.86	7.25 ± 1.73 ^b^	69.47 ± 4.38	9.32 ± 0.67	20.47 ± 7.80	3.66 ± 1.76	109.79 ± 32.44 ^a,b^	6.79 ± 2.94 ^b^	2.47 ± 0.22	2663 ± 2054 ^a^
	RA+EG/AC	205.71 ± 25.13	6.00 ± 2.24	70.37 ± 4.33	8.88 ± 1.18	24.40 ± 12.46	3.10 ± 1.59	140.59 ± 16.11	4.96 ± 1.07 ^b^	2.49 ± 0.14	15,208 ± 8298
**SHRs**	VEH	236.00 ± 41.59	6.20 ± 1.30	65.58 ± 2.29	10.16 ± 0.64	33.08 ± 8.27	2.22 ± 0.13	157.93 ± 3.95	4.39 ± 0.46	2.43 ± 0.22	11,185 ± 3272
	VEH+EG/AC	221.67 ± 7.60	5.67 ± 0.38	67.59 ± 2.30	9.73 ± 0.65	28.42 ± 7.80	2.28 ± 0.56	151.12 ± 5.79	4.70 ± 0.53	2.51 ± 0.29	15,057 ± 6863
	HCTZ+EG/AC	224.00 ± 11.01	5.20 ± 0.42 ^b^	63.72 ± 5.55	9.82 ± 0.46	30.12 ± 4.23	2.17 ± 1.26	166.35 ± 7.16 ^a^	4.43 ± 0.54	2.43 ± 0.25	12,158 ± 2727
	RA+EG/AC	240.00 ± 12.64	6.33 ± 0.75	70.24 ± 1.99^b^	9.57 ± 0.54	29.36 ± 3.32	2.10 ± 0.47	146.08 ± 13.63	4.69 ± 0.58	2.74 ± 0.21	10,335 ± 1281

Values expressed as mean ± standard deviation; ^a^: *p* < 0.05 compared with the VEH+EG/AC group; ^b^: *p* < 0.05 compared with the VEH group.

**Table 3:** Renal oxidative stress and inflammation parameters.

	Group	LPO mmol/mg Tissue	GSH µg/g Tissue	GST µmol/min/mg pt	SOD U/mg pt	CAT µmol/min/mg	NAG mD.O/mg pt	MPO mD.O/mg pt
**NTRs**	VEH	4.53 ± 0.26	1267.16 ± 92.48	0.44 ± 0.30	4.32 ± 0.84	59.48 ± 12.50	113.75 ± 20.93	5.26 ± 1.04
	VEH+EG/AC	4.32 ± 0.13	1524.96 ± 131.45	0.45 ± 0.20	4.98 ± 1.68	19.94 ± 5.93 ^b^	83.04 ± 14.76 ^b^	3.54 ± 1.02
	HCTZ+EG/AC	4.61 ± 0.20	1705.35 ± 99.57 ^b^	0.23 ± 0.01	5.83 ± 0.47	40.03 ± 2.89 ^a,b^	75.35 ± 10.11 ^b^	3.00 ± 0.25 ^b^
	RA+EG/AC	4.39 ± 0.28	1480.76 ± 258.07	0.33 ± 0.23	4.92 ± 1.15	26.76 ± 7.92 ^b^	89.29 ± 15.24	3.65 ± 1.47
**SHRs**	VEH	4.45 ± 0.29	1111.49 ± 71.06	0.30 ± 0.08	3.55 ± 0.51	35.54 ± 17.74	82.16 ± 15.62	3.11 ± 0.55
	VEH+EG/AC	4.53 ± 0.34	1410.36 ± 129.83 ^b^	0.29 ± 0.11	4.25 ± 1.49	13.29 ± 12.26	109.86 ± 31.41	4.01 ± 1.43
	HCTZ+EG/AC	4.17 ± 0.15	741.68 ± 145.62 ^a,b^	0.73 ± 0.33 ^a,b^	5.11 ± 0.80	41.23 ± 12.31	100.97 ± 26.67	4.87 ± 1.59
	RA+EG/AC	4.42 ± 0.20	1261.86 ± 129.83	0.70 ± 0.24 ^a,b^	5.75 ± 0.81^b^	57.89 ± 20.50^a^	117.88 ± 23.05	5.16 ± 1.59

Values expressed in mean ± standard deviation; ^a^: *p* < 0.05 compared with the VEH+EG/AC group; ^b^: *p* < 0.05 compared with the VEH group.

**Table 4:** Liver oxidative stress and inflammation parameters.

	Group	LPO mmol/mg Tissue	GSH µg/g Tissue	GST µmol/min/mg pt	SOD U/mg pt	CAT µmol/min/mg	NAG mD.O/mg pt	MPO mD.O/mg pt
**NTRs**	VEH	3.89 ± 0.04	1043.98 ± 75.66	0.79 ± 0.14	4.67 ± 0.34	7.30 ± 6.05	96.24 ± 17.48	5.44 ± 0.68
	VEH+EG/AC	3.88 ± 0.10	749.56 ± 145.98 ^b^	0.29 ± 0.19 ^b^	3.17 ± 0.75 ^b^	23.07 ± 9.63 ^b^	52.29 ± 10.93 ^b^	4.43 ± 0.87
	HCTZ+EG/AC	4.07 ± 0.13 ^a,b^	1188.18 ± 205.62 ^a^	0.42 ± 0.13 ^b^	3.90 ± 0.33	21.79 ± 4.31	61.58 ± 6.44 ^b^	3.93 ± 0.81 ^b^
	RA+EG/AC	3.61 ± 0.12 ^a,b^	1343.79 ± 222.12 ^a,b^	0.45 ± 0.17 ^b^	4.59 ± 0.68 ^a^	26.30 ± 9.40 ^b^	55.04 ± 11.16 ^b^	4.47 ± 0.89
**SHRs**	VEH	4.14 ± 0.31	1318.91 ± 244.04	0.62 ± 0.18	3.43 ± 0.32	4.58 ± 2.60	61.84 ± 12.11	5.17 ± 0.94
	VEH+EG/AC	3.80 ± 0.14 ^b^	2385.20 ± 298.30 ^b^	0.70 ± 0.15	3.86 ± 0.50	19.41 ± 7.65 ^b^	61.68 ± 7.80	5.19 ± 1.03
	HCTZ+EG/AC	3.71 ± 0.17 ^b^	1682.13 ± 392.97 ^a^	0.37 ± 0.21 ^a^	5.79 ± 1.94 ^a,b^	9.01 ± 4.83	65.13 ± 14.47	6.38 ± 2.30
	RA+EG/AC	3.73 ± 0.19 ^b^	1854.99 ± 357.46	0.39 ± 0.16 ^a^	4.51 ± 1.15	18.30 ± 7.59 ^b^	56.38 ± 4.99	4.58 ± 0.60

Values expressed in mean ± standard deviation; ^a^: *p* < 0.05 compared with the VEH+EG/AC group; ^b^: *p* < 0.05 compared with the VEH group.

**Table 5:** Predicted binding energies of rosmarinic acid and predicted H-bonds to the enzymes.

Enzymes	PDB Code	RA Binding Affinity (kcal/mol)	H-Bonds
Human glycolate oxidase	2RDT	−9.5	Tyr27, His260, Ala81, Lys236, Arg295
MMP-2 catalytic domain	1HOV	−7.0	Ala84, Tyr142, Glu121
MMP-9 catalytic domain	4XCT	−10.5	Ala189, Glu227, His226, Met247, Gly186
Phosphoethanolamine cytidylyltransferase	3ELB	−9.8	Phe222, His229, Ala219, Gly308, Ser336, Leu340, Glu311, Tyr290

## Data Availability

Data will be made available on reasonable request.
